# A miRNA Target Prediction Model Based on Distributed Representation Learning and Deep Learning

**DOI:** 10.1155/2022/4490154

**Published:** 2022-07-25

**Authors:** Yuzhuo Sun, Fei Xiong, Yongke Sun, Youjie Zhao, Yong Cao

**Affiliations:** ^1^College of Big Data and Intelligent Engineering, Southwest Forestry University, Kunming, China; ^2^College of Material Science and Engineering, Southwest Forestry University, Kunming, China

## Abstract

MicroRNAs (miRNAs) are a kind of noncoding RNA, which plays an essential role in gene regulation by binding to messenger RNAs (mRNAs). Accurate and rapid identification of miRNA target genes is helpful to reveal the mechanism of transcriptome regulation, which is of great significance for the study of cancer and other diseases. Many bioinformatics methods have been proposed to solve this problem, but the previous research did not further study the encoding of the nucleotide sequence. In this paper, we developed a novel method combining word embedding and deep learning for human miRNA targets at the site-level prediction, which is inspired by the similarity between natural language and biological sequences. First, the word2vec model was used to mine the distribution representation of miRNAs and mRNAs. Then, the embedding is extracted automatically via the stacked bidirectional long short-term memory (BiLSTM) network. By testing, our method can effectively improve the accuracy, sensitivity, specificity, and *F*-measure of other methods. Through our research, it is proved that the distributed representation can improve the accuracy of the deep learning model and better solve the miRNA target site prediction problem.

## 1. Introduction

MircroRNAs (miRNAs) are small single-stranded RNA molecules with a length of 22 nucleotides (nts), which are widely found in eukaryotes [1]. Mature miRNAs combine with proteins to form RNA-induced silencing complexes (RISC) that cause mRNA hydrolysis or inhibit translation by binding to the target sites of mRNA [2]. The miRNAs regulate more than 60% of protein-coding genes in humans and other mammals and play crucial roles in many biological processes, including cell development, differentiation, proliferation, and apoptosis [3]. Past evidence has shown that miRNAs are also closely associated with diseases such as cancer and metabolic abnormalities [4, 5]. However, up to now, the functions of a large number of miRNAs are still unclear. Therefore, finding the target sites of miRNA is of great significance for understanding its function and regulatory mechanism.

For miRNA target gene research, there are currently three types of methods that can effectively find the target sites of miRNA, but there are still some problems to be improved. The method based on the biological experiment [6] can find target genes accurately, but the artificial experiment is time-consuming and expensive. Although the method based on database [7] search and matching can get the result quickly but is inaccurate, it cannot determine the information not included in the database. A result of the above method problems prompted the development of machine learning algorithm tools. With the continuous development of artificial intelligence technology, most of the recent methods are based on deep learning models have a great result. DeepTarget [8] is an end-to-end model at the two levels of processing site and gene. The feature extraction of miRNA and mRNA is carried out by autoencoder, respectively, and then uses gate recurrent unit (GRU) to learn the sequence-to-sequence interactions between miRNA and their targets. DeepMirTar [9] uses 750 manually extracted features in 7 categories, using a stack denoising autoencoder and achieves 93% accuracy at the site level. Xueming et al. [10] use a multilayer convolutional neural network (CNN) stack structure, processing site, and gene levels prediction, and the model can use full-length mRNAs as input. However, even though these deep learning methods have achieved good results, there are still some problems that need to be improved. In the past, the traditional methods used the simulation method to generate negative class data [8, 9], which would increase the probability of false-positive and the poor generalization ability [11]. In recent years, some studies add more and more artificial features, even deep learning-based methods [9], but feature engineering is time-consuming and laborious and may bring in subjective influence factors. Moreover, most of the past methods used one-hot coding [8, 11], which treated the nucleotide sequence as a series of meaningless letters without the biological significance of the sequence.

To solve the above problems, a novel end-to-end target gene prediction method at the site level is proposed in this paper. From a new perspective, based on the similarity between biological sequences and natural languages, a neural network is used to learn distribution representation of miRNA and mRNA sequences [12]. In our method, miRNA and mRNA were processed as two different languages, and the nucleotides in the sequence were treated as words, referring to the word embedding method commonly used in natural language processing (NLP) tasks. Compared with the one-hot representation, the distributed representation uses a dense vector to represent nucleotides, which can describe the similar relationship between nucleotides to a certain extent, and thus obtain sequence embedding with more information. In addition, the positive and negative class sample data used in this paper are all verified by a variety of experiments to avoid the problems caused by the mock data in the past. The original sequence data is processed by the word embedding model and directly entered into the neural network for feature extraction and final classification, omitting the manual feature engineering steps. Finally, through 5-fold cross-validation, our method outperforms other commonly used advanced database-based and deep learning methods on the two data sets.

## 2. Materials and Methods

### 2.1. Data Description

The prediction of miRNA target genes can be divided into site-level and gene-level, and the main difference lies in the different data. The method proposed in this paper deals with site-level prediction, the data included miRNA sequences and candidate target sites (CTS), and the sample was labeled binding or not. The dataset used in the experiment are all from public databases, and the positive and negative pairs have been verified via biological experiments. We utilize experimental negative data instead of mock ones, and the problem of high false positives in the current prediction model can be solved. The dataset for this experiment consists of two public databases that have been used in recent studies [11]. Diana-Tarbase [13] provided experimentally verified miRNA-mRNA interaction information, including 121,090 positive and 2940 negative pairs. Mirtarbase [14] provides 410,000 positive pairs of miRNA-mRNA interactions. Through screening and deleting data with contradictory results in different experiments and merging duplicate data, the gene-level matching data of 151956 positive and 548 negative pairs verified by many kinds of experiments were finally obtained. According to the biological characteristics of miRNA-mRNA binding, multiple miRNAs can bind to one mRNA at the same time, and one miRNA can also bind to multiple CTS. Therefore, although there are only 548 negative samples at the gene level, by screening out CTS at the gene level, we can finally get samples with relatively equal positive and negative classes.

To make a site-level miRNA and CTS pairing information dataset, two databases, PAR-CLIP [15] and CLASH [16], should be utilized to provide the site-level pairing information of miRNA-mRNA verified by experiments. The positive pairs that form stable duplexes, namely, those have negative free energy based on ViennaRNA [17], are remained and complemented by including broadly conserved sites from TargetScanHuman database [18]. Similarly, the negative pairs that have length of up to 30 nts and form stable duplexes are considered as experimentally verified negative pairs. As the result, 33,142 site-level positive and 32,284 site-level negative pairs are used to train the proposed approach.

### 2.2. Distribution Representation of miRNA and mRNA Sequences

**Figure 1:**
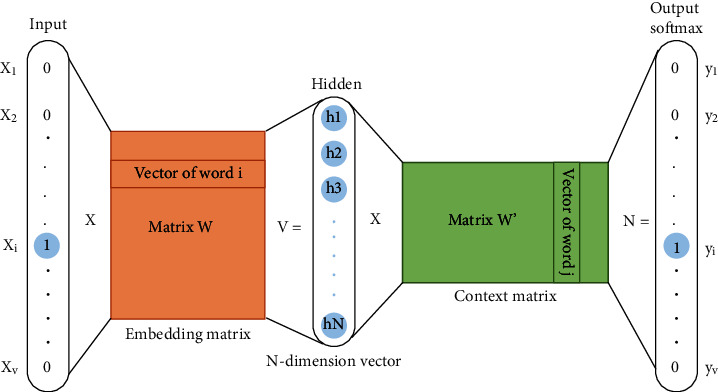
The skip-gram model structure. Skip-gram is trained by predicting words surrounding the central word, after training, the weights matrix *W* of the hidden layer is obtained, that is, word vectors.

To obtain the distribution and expression of miRNA and mRNA, we use the mature miRNA and mRNA sequences of the human genome as corpus, and word2vec [19, 20] is used for training, respectively. There are two network structures of skip-gram and Bag-of-Words in word2vec. Since it has been found in some studies that skip-gram has better results, this article uses the skip-gram model, which predicts the words around the input word. We treat nucleotides as words, and word2vec with the skip-gram [21] model acquires a distributed representation for each nucleotide by training the three layers neural network, as shown in Figure 1. For example, if a sequence consists of T nucleotides, *w*_*t*_ stands for the *t*-th nucleotide, and the model predicts the nucleotides appearing near *w*_*t*_. For a given sequence (*w*_1_, *w*_2_,…, *w*_*t*_), the goal of training model is to maximize the mean log probability:

(1) $$\begin{array}{c} \max\frac{1}{N}\overset{N}{\underset{n-1}{\sum }}\underset{-c\leq m\leq c,m\neq 0}{\sum }\log P\left(w_{n+m}\left|w_{n}\right.\right). \end{array}$$

**Figure 2:**
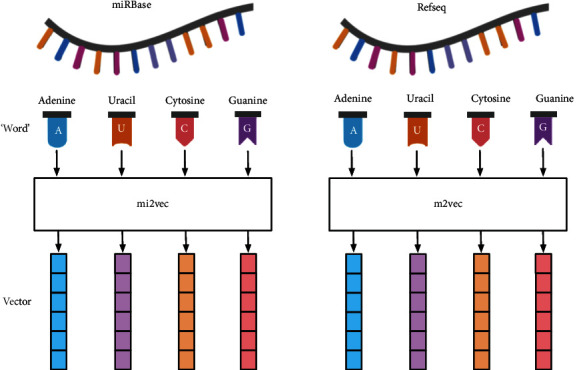
The procedure for training mi2vec and m2vec. The corpus of miRNA and mRNA sequences obtained from miRBase and Refseq. Treat each miRNA and mRNA sequence as a special sentence, and treat the nucleotides as the words that make up the sentence. Use the skip-gram model to train a vocabulary list composed of nucleotides, and get the vectorized representation of the nucleotides.

*c* stands for the distance to the central word; the log probability distribution can be defined as follows:

(2) $$\begin{array}{c} \log P\left(w_{0}\left|w_{t}\right.\right)=\log\frac{e^{v\prime_{w_{0}}^{T}v_{w_{t}}}}{\sum_{w=1}^{W}e^{v\prime_{w}^{T}v_{w_{t}}}}, \end{array}$$

where the *v*_*w*_ and *v*′_*w*_ are the input and output vector of nucleotide *w*, respectively. *W* is the size of training miRNA or mRNA training lexicon. In recent bioinformatics studies, some methods [22, 23] have been used to train word embedding models for DNA, protein, and lncRNA, and it has been proved that this method is superior to the traditional processing sequence embedding methods such as one-hot and K-mers. In this paper, the word2vec tool in the Gensim package [24] was used for pretraining. The training process of the skip-gram model was improved, and negative sampling and hierarchical softmax methods were used to improve the training speed. The training data are derived from miRBase [25] and Refseq [26] databases, which store the most authoritative and complete relevant sequence data at present. The overall sequence embedding process is shown in Figure 2, where the training process of miRNA and mRNA is named mi2vec and m2vec.

The embedding dimension is considered to be the most important hyperparameter parameter [27] in NLP, so vector_size = 2, 4, 10, 20, 30, 50, and 100 different output dimensions are set to facilitate the comparison and selection of the optimal parameters in subsequent experiments. The Gensim package parameters of the model are min_count = 1, window = 5, and epoch = 10. Where window stands for *c* in the previous article, and the epoch is the count of iterations over the corpus. When the min_count (means minimum word frequency) is set too high, the model only counts high-frequency words, which is not conducive to learning discriminative word vectors from sequence representation. Other parameters are default.

### 2.3. Deep Learning Method

**Figure 3:**
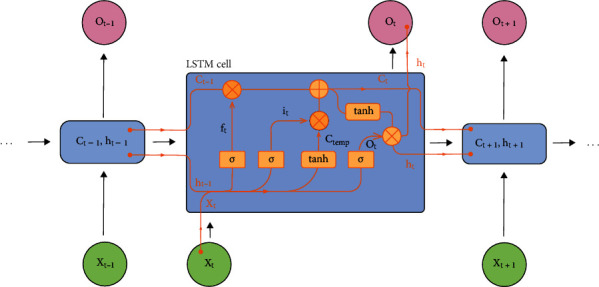
The structure of LSTM-cell.

Long short-term memory (LSTM) is an artificial recurrent neural network (RNN) architecture used in the field of deep learning and is composed of the forget gate, the input gate, and the output gate. The cell remembers values over arbitrary time intervals and the three gates regulate the flow of information into and out of the cell.  Figure 3 shows LSTM cell structure.

The state of forget gate is related to the output of last cell *h*_*t*−1_:

(3) $$\begin{array}{c} f_{t}=\sigma \left(W_{f}•\left[h_{t-1},x_{t}\right]+b_{f}\right), \end{array}$$

where *σ* is the sigmoid function, and *x*_*t*_ represents the cell input of time *t*. It is the same for *i*_*t*_, and the only difference is that the weight matrix is *W*_*i*_ and bias is *b*_*i*_. And for the cell state *C*_temp_, the *σ* function is replaced by tanh and also with a *W*_*c*_ and *b*_*c*_.

(4) $$\begin{array}{c} i_{t}=\sigma \left(W_{i}•\left[h_{t-1},x_{t}\right]+b_{i}\right), \end{array}$$

(5) $$\begin{array}{c} c_{\text{temp}}=\tanh\left(W_{c}•\left[h_{t-1},x_{t}\right]+b_{c}\right). \end{array}$$

And the new cell state *c*_*t*_ can be calculated in equation:

(6) $$\begin{array}{c} c_{t}=f_{t}•c_{t-1}+i_{t}•c_{\text{temp}}. \end{array}$$

The output *o*_*t*_ and hidden state *h*_*t*_ for this moment can be calculated as follows:

(7) $$\begin{array}{c} o_{t}=\sigma \left(W_{o}•\left[h_{t-1},x_{t}\right]+b_{o}\right), \end{array}$$

(8) $$\begin{array}{c} h_{t}=o_{t}•\tanh\left(c_{t}\right). \end{array}$$

LSTM networks are well-suited to classifying, processing, and making predictions based on time series data since there can be lags of unknown duration between important events in a time series, and it can deal with the vanishing gradient problem that can be encountered when training traditional RNNs. Because of its design characteristics, LSTM is very suitable for processing text and biological data.

However, it is still impossible to encode information from back to front when using LSTM to model the sequence. BiLSTM consists of two LSTMs: one taking the input in a forward direction, and the other in a backward direction. BiLSTM can obtain the information of the two directions of the positive sequence and the reverse sequence of the sequence and then combine the output (e.g., knowing what nucleotides immediately follow and precede a nucleotide in a sentence).

As shown in Table 1, through experimental comparison of basic RNN, GRU, LSTM, and BiLSTM, the accuracy of LSTM in the model test is higher than that of other structures, bidirectional and unidirectional tests are conducted in two-layer stacked LSTM, respectively, and it is finally proved that BiLSTM can achieve better results. We used tensorboard tool to record model parameters, and weights, parameters, layers, and other information can be referred to supplementary file Figure S1 and S2.

### 2.4. Overall Workflow

**Figure 4:**
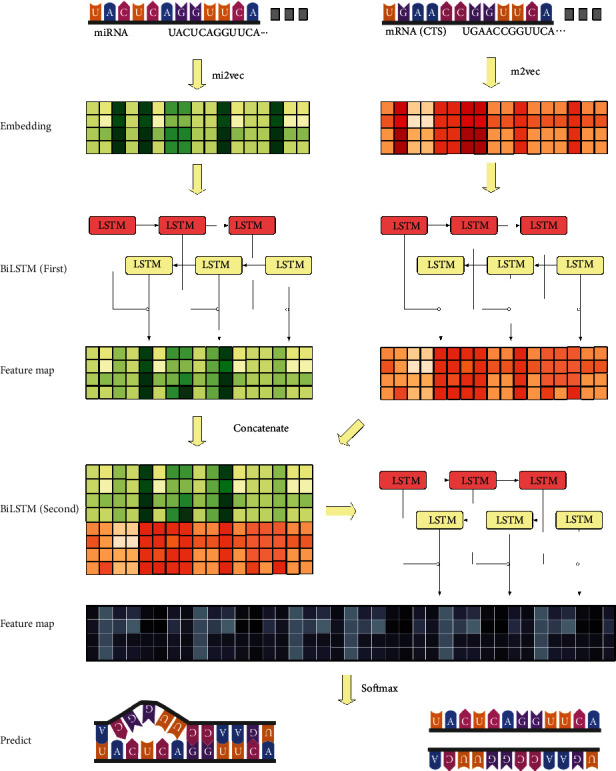
The overall workflow. Fill the input miRNA and CTS original sequence to a uniform length, and then replace each nucleotide in the sequence with the vector trained by mi2vec and m2vec. The 50-dimension embedding of miRNA and CTS passes through the first BiLSTM layer, respectively, and then concatenates the outputs feature maps, then passes through the second BiLSTM get to 200-dimensions, and finally, uses the linear layer to reduce the dimension to 2-dimensions and prediction by softmax.

Figure 4 shows an overview of the method proposed in this paper. First, the original sequence data of miRNA and CTS were processed in a uniform length. According to the maximum length of human mature miRNA is 26 nts, the seed region binding to CTS is usually the 2-8 nucleotide site [1], so the input sequence was all filled to 30 nts.

Through the vector representation of each base obtained through the mi2vec and m2vec processes in the previous chapter, each nucleotide in the input sequence is replaced with a vector, and our input data is finally converted into a 50-dimensional matrix.

In many NLP studies [28, 29], the method of word embedding combined with RNN has a breakthrough performance. According to the experimental results of different RNN structures in the last chapter, we adopt the BiLSTM for feature extraction. Use BiLSTM to extract the sequence features of miRNA and CTS, respectively. Then, to model interactions between miRNA and CTS, the feature map output by the first layer of BiLSTM is concatenated into one tensor. In this way, two layers of stacked BiLSTM have the advantage of learning both the intrinsic spatial and sequential features of miRNA and CTS. For details about the variation of the tensor dimension, see Figure S1.

miRNA target site prediction can treat the target as a dichotomous problem to determine whether miRNA binds or not to CTS, and the sample label indicates whether binding occurs. After the feature extraction via stacked BiLSTM, the feature dimension is gradually reduced to 2-dimensional output by using two linear layers. Finally, the combination relationship was determined by the softmax function.

## 3. Results and Discussions

In this study, a novel miRNA and CTS interaction prediction model based on sequence distributed representation and deep learning is proposed. In this chapter, the following experiments are designed to verify the performance of the model. First, the effects of one-hot and word2vec coding on the accuracy of deep learning models were compared, and the sequence of distributed representation is visualized and analyzed. Then, a variety of evaluation indexes will be used to verify the performance of the model and compared with other target prediction methods. Finally, each result of this experiment is discussed in depth.

### 3.1. Impact of Distributed Representation on Model

In this paper, one-dimensional convolutional neural networks (CNN1d) and BiLSTM, two models which are good at processing sequence data, are selected to test study the influence of the data embedding method on the accuracy of neural networks. One-hot coding and different dimensions of word2vec output were used in the comparison experiment. For training, we optimized the weighted cross-entropy loss function using Adam optimizer (batch size: 32, the number of epochs: 100). The remaining hyperparameters used were set to be the default PyTorch implementation, and the accuracy on the test set was recorded.

**Figure 5:**
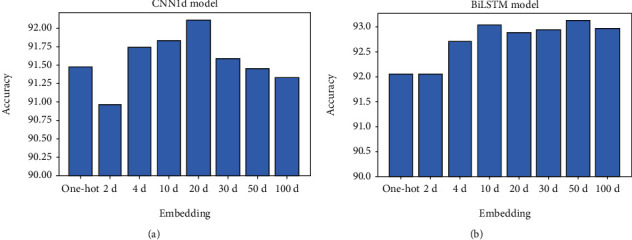
Different embedding influences the deep-learning model on accuracy. (a) Shown is the use of one-hot and word2vec (2, 4, 10, 20, 30, 50, and 100-dimensions) as the input of the CNN1d model. (b) The same test using the BiLSTM model.

It can be seen from Figure 5 that the word2vec method can effectively improve the accuracy of the two deep learning models of CNN1d and BiLSTM. The results show that the method of word embedding is equally effective for biological sequences of miRNA and mRNA. Additionally, we also tested the one-hot encoding as the input of the normal LSTM model, and the result was 92.1% accuracy.

Biological sequences vectorized by representation learning can be directly used for biological tasks, such as function and structure prediction. Such as if the vector similarity between proteins or RNAs is high, it can be inferred that they possess similar functions and structures. The vector similarity/distance can be calculated using linear algebra operations, such as dot product, Euclidian distance, and cosine similarity.

**Figure 6:**
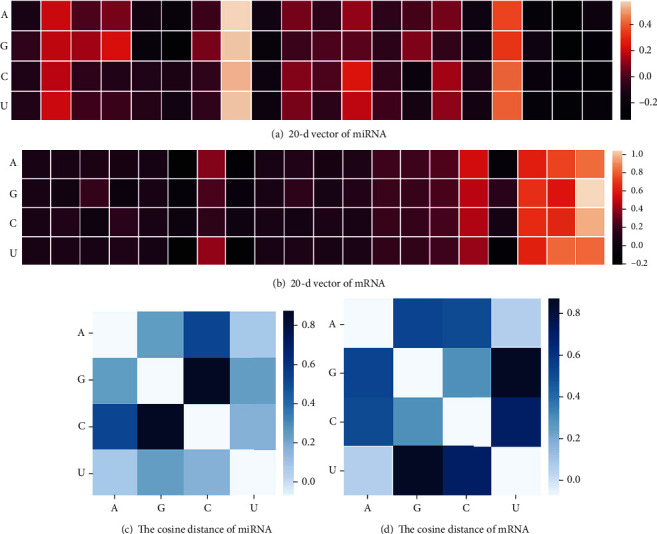
Visualization analysis of miRNA and mRNA. (a) The nucleotide 20 dimension vector representation of miRNA. (b) The nucleotide 20 dimension vector representation of mRNA. (c) The cosine distance between miRNA nucleotides was calculated based on the 20-dimensional vector. (d) The cosine distance between mRNA nucleotides was calculated based on the 20-dimensional vector.

In order to explore the embedding meaning of miRNA and mRNA sequences, the 20-dimensional vector with the best performance on CNN1d was selected as an example for visualization research, and the similarity between nucleotides was analyzed through cosine distance calculation. As can be seen from the visualization analysis of miRNA and mRNA, Figures 6(a) and 6(b) are visualized heat maps of miRNA and mRNA distributed as 20-dimensional vectors, respectively, and it can be seen from the diagram that the representation of each nucleotide is different. By calculating the cosine distance of the vector, it can be seen from Figures 6(c) and 6(d) that the similarity between the nucleotides in miRNA and mRNA is not the same. The smaller the distance, the more similar the nucleotides. Particularly, the successful encoding of words via representation learning has been recognized as an essential research area because the performance of NLP and deep learning depends on the quality of the representation. Thus, a good representation of a biological sequence is critical for clustering, function, structure, and disorder prediction.

### 3.2. Parameters' Effect on the Model

The training of the neural network model is determined by its own structural parameters and hyperparameters. In order to obtain the model with optimal performance, the following experiments are designed to determine the model parameters. First, we will compare each layer output unit of BiLSTM and linear layers in order to determine the best model structure. Second, we adjust for the hyperparameters of the training and study the influence of hyperparameters on the experimental results.

#### 3.2.1. Adjusting Structural Parameters

Adjust our model structure according to previous methods [30]. Input dimension is represented by a 50-dimension vector of mi2vec and m2vec according to the experimental results of 3.1, so the input dimension is 50, and the output dimension is also set at 50. In order to verify the previous theory and reference comparison, the first layer BiLSTM set hidden size value (16, 32, 50, 64, 128) for the experimental test, the hyperparameters are temporarily set: batch size = 32, lr = 0.001, and the optimizer uses Adam.

The adjustment results of the model structure are shown in Table 2. After trying 10 model structures, 50-32-32-2 is finally determined, where the value represents the number of output units of each layer of the network.

#### 3.2.2. Adjusting Hyperparameter

Hyperparameters play an important role in the training model. Typical hyperparameters include lr, batch size, and the optimizer. We use the usual method of adjusting the hyperparameters: fix all the hyperparameters and then try to modify one of them. Adam has excellent performance and is the most widely used in today's deep learning models. In addition, an attempt to use a stochastic gradient descent (SGD) optimizer failed to converge the loss function in this experiment, so Adam is determined to be used as the optimizer. Then, adjust lr and batch size, respectively, and the results are shown in Table 3.

It can be seen from the results that the model is sensitive to hyperparameters, and some wrong settings will make the model unable to converge. By comparison, the model achieved the highest accuracy when lr = 0.001. Although the selection of batch size has little effect on the results, the larger batch size can significantly reduce the training time. So we made a trade-off and set the batch size = 32.

### 3.3. Contrast Experiment

We selected the optimal parameters through a series of experiments mentioned above. In addition, the miRNA and mRNA sequence data were cleaned, and the unverified sequences were removed and the pretraining again. Finally, our model is compared with some site prediction methods.

#### 3.3.1. Evaluation Indicators

We use cross-validation for testing. Record test results using a confusion matrix. As a dichotomous problem of miRNA target gene site prediction, accuracy (Acc), sensitivity (Sens), specificity (Spec), and *F*-measure are commonly used as evaluation indexes of the comprehensive performance of the prediction model [11, 31, 32]. The calculation formula is as follows:

(9) $$\begin{array}{c} \text{Acc}=\frac{\text{TN}+\text{TP}}{\text{TN}+\text{TP}+\text{FN}+\text{FP}}, \end{array}$$

(10) $$\begin{array}{c} \text{Sens}=\frac{\text{TP}}{\text{TN}+\text{FN}}, \end{array}$$

(11) $$\begin{array}{c} \text{Spec}=\frac{\text{TN}}{\text{TN}+\text{FP}}, \end{array}$$

(12) $$\begin{array}{c} F-\text{measure}=\frac{2\text{TP}}{2\text{TP}+\text{FP}+\text{FN}}. \end{array}$$

According to the definition of confusion matrix of dichotomy, TP, FP, TN, and FN represent true positive, false positive, false negative, and true negative, respectively. Also, when we compare the predictive performance between methods, perform some statistical tests, calculating *p* values (*p* < 0.0001; Student's *t*-test).

#### 3.3.2. Performance Comparison of Two Datasets and Different Methods

In order to prove the generalization ability of our model, the 5-fold cross-validation method was used to randomly divide the original dataset into five folds, of which four folds were used as training and one fold as a test. The design experiment was compared with the current commonly used database prediction tools and deep learning target site prediction methods. These include two databases, PITA and TargetScan [33, 34], and two deep learning methods, CNN1d and DeepMirTar [9, 11]. The DeepMirTar and the data set used in this paper were, respectively, used for comparative experiments. DeepMirTar uses 750 features to train their model, but does not provide 750 features and does not support input of other data, so we only use the dataset for comparison and cannot report the performance of the model on experimented data. The results and comparison methods are shown in Table 4.

From the comparative experimental results, it can be seen that on the dataset used in the DeepMirTar paper, our method achieves better results than the DeepMirTar method. Moreover, in this paper, the neural network is used to automatically extract features instead of manual feature engineering, which greatly reduces the complexity, saves a lot of time, and has higher accuracy. The negative pairs in the dataset used in this paper have been verified by a variety of experiments to replace the mock data used by DeepMirTar. In comparison with database methods, the data verified by experiments has higher specificity, which can reduce the probability of false positive in the prediction. Finally, for the experimental data used in this paper, through word2vec pretraining data, combined with the BiLSTM model, and compared with one-hot coding and CNN1d model, our method has been improved in Acc, Sens, Spec, *F*-measure, and *p* value.

**Figure 7:**
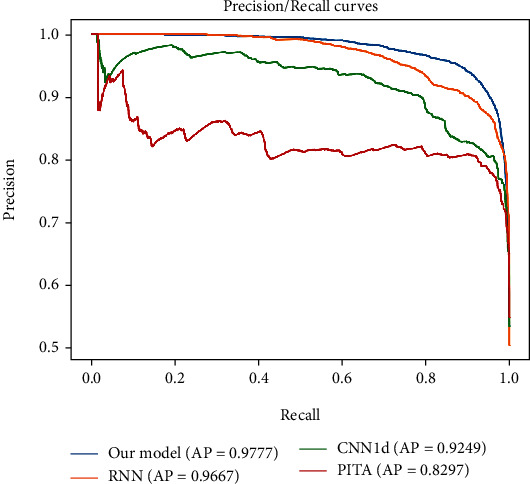
The PR curves. The PR curves and APs of our model and other models.

To further evaluate our model, we plotted the prediction and recall (PR) and ROC curves of prediction on the test dataset. PR curves and average precision (AP) often quantify retrieval efficacy in general information retrieval. The PR curve was plotted based on the prediction results of precision and recall. The average precision was calculated and shown in Figure 7. Our model outperforms other methods with an average accuracy of 97.77%.

**Figure 8:**
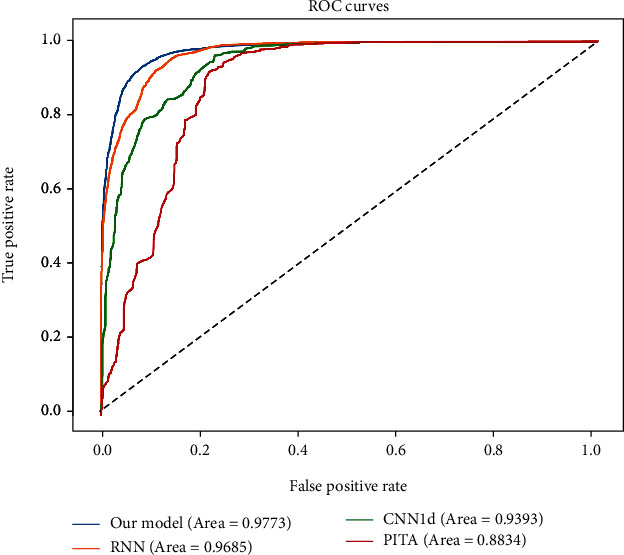
The ROC curves. The ROC curve was plotted based on the prediction results of the test dataset. The area under the ROC curve was calculated.

Also, we plotted the receiver-operating characteristic curve (ROC) with the calculated area under the ROC curve (AUC). With decreasing thresholds on the decision function used, corresponding false positive rates (FPR) and true positive rates (TPR) were computed. ROC curve was drawn based on a series of FPR and TPR. As shown in Figure 8, the AUC of ROC curve is 97.73%, indicating high performance for recognizing the target site of the miRNAs.

### 3.4. Discussion of the Biological Significance of the Model

In recent years, studies have shown that miRNAs can be used for the diagnosis, treatment, and prognosis of cancer patients [35]. miRNAs that are upregulated in cancer cells and promote carcinogenesis by suppressing tumor suppressor genes are considered oncogenic miRNAs, while downregulated miRNAs that typically prevent cancer development by inhibiting proto-oncogene expression are known as tumor suppressor miRNAs. Through miRNA and mRNA binding sites, efficient miRNA mimics can be designed and synthesized for proto-oncogenes or tumor suppressor genes.

When conditions permit, RT-qPCR technology [36] is often used for experimental detection, but for researchers with limited funds and time, reliable bioinformatics tools are needed to predict miRNA target sites. On the other hand, the new method proposed in this paper is an end-to-end model combining representation learning and deep learning. Representation learning can automatically learn deeper feature information from raw data. The model can directly use the original sequence as input and finally get the prediction result. It is no longer necessary to rely on human experience to design features from data, which greatly reduces the difficulty of model design and use. For biological researchers, the use of this model does not require prior knowledge of machine learning feature extraction and only relies on bioinformatics knowledge to assist in target site prediction.

## 4. Conclusions

miRNA is an indispensable component of complex transcriptome regulation, which affects life processes and related diseases. To study the function and mechanism of miRNA, the determination of miRNA binding sites is the primary goal. In this study, we developed a deep learning method for predicting miRNA target site by pretraining distribution representation model, using skip-gram word embedding model and human genome-wide miRNA and mRNA sequences. By comparing the performance of different coding methods and other prediction methods, the results prove the effectiveness of the proposed method.

Through other recent literature and our research, it is proved that the NLP method is effective and feasible to deal with the biological sequence problem. The experiment proved this feature extraction scheme works well. However, word2vec has certain limitations. Since there is a one-to-one correspondence between words and vectors, the polysemy problem cannot be solved. For example, bases or subsequences at different positions in a sequence represent different biochemical and biophysical significance. Word2vec is a static method. Although it is general, it cannot be dynamically optimized for specific tasks. So we can try to experiment with dynamic embedding models in the future. Although NLP-based biological sequence analysis is in its early stages and warrants further development, in the light of novel challenges in biology, such as single-cell analysis, genome design, and epigenetic regulation research, representation learning may contribute to the progression of bioinformatics studies, thus revealing the grammar of life. With the continuous development of NLP models, more advanced NLP models can be tried to deal with biological sequences and solve biological problems in the future.

## Figures and Tables

**Table 1:** The two layers of RNN selected different architectures for comparison.

First layer	Second layer	Accuracy (%)
RNN	RNN	90.93
GRU	GRU	92.96
LSTM	LSTM	93.16
LSTM	BiLSTM	92.87
BiLSTM	LSTM	93.23
BiLSTM	BiLSTM	93.45

**Table 2:** Model structure comparison.

First BiLSTM	Second BiLSTM	First linear	Second linear	Accuracy (%)
8	32	32	2	92.23
16	32	32	2	92.72
32	32	32	2	93.01
50	32	32	2	93.34
64	32	32	2	92.75
128	32	32	2	92.17
50	8	8	2	92.89
50	16	16	2	92.58
50	64	64	2	92.48
50	128	128	2	92.75

**Table 3:** Model hyperparameter comparison.

Optim	lr	Batch size	Accuracy (%)
Adam	0.0005	32	92.5
	0.001	32	93.34
	0.005	32	91.23
	0.01	32	50.01
	0.001	8	92.11
	0.001	16	92.96
	0.001	64	93.02
	0.001	128	92.53

**Table 4:** Performance evaluation metrics on miRNA target sites prediction.

Dataset	Method	Acc (%)	Sens (%)	Spec (%)	*F*measure (%)	*p* value ^b^
Mock	TargetScan	58.01	60.23	59.22	59.72	*p* < 10^−6^
	PITA	49.81	58.72	40.82	48.16	*p* < 10^−6^
	DeepMirTar	93.48	92.35	94.79	93.55	*p* < 10^−4^
	Our method ^a^	96.86	96.97	96.75	96.91	—
Experimented	TargetScan	55.77	39.45	72.08	47.12	*p* < 10^−6^
	PITA	50.53	13.65	87.41	21.62	*p* < 10^−5^
	CNN1d ^a^	91.05	94.06	87.96	91.40	*p* < 10^−3^
	Our method ^a^	96.04	95.65	96.44	96.09	—

a: 5-fold cross-validation results mean. b: the *p* value according to Student *t*-test (Acc) and indicating comparison between the our model and other methods in experimented test dataset.

## Data Availability

Our study is based on open-source data, and thus, there are no ethical issues or conflicts of interest. The data used to support the findings of this study are available from the corresponding author upon request.

## Supplementary Materials

Supplementary Materials We used TensorBordX to record all parameter changes that the model went through from input to output, where RNN represents our deep learning model, LSTM (rnn1) represents the first layer of BiLSTM, LSTM (rnn2) represents the second layer of BiLSTM, Linear (fc1) represents the first linear layer, and Linear(fc2) represents the second linear layer. Figure S1: where RNN represents our deep learning model, LSTM (rnn1) represents the first layer of BiLSTM, LSTM (rnn2) represents the second layer of BiLSTM, Linear (fc1) represents the first linear layer, and Linear(fc2) represents the second linear layer. Figure S2: details of the internal parameters of the BiLSTM structure.
