# Supplementary figures and images for: A miRNA Target Prediction Model Based on Distributed Representation Learning and Deep Learning
Source: Comput Math Methods Med. 2022 Jul 25;2022:4490154. doi: 10.1155/2022/4490154 (PMC9343202; doi:10.1155/2022/4490154)

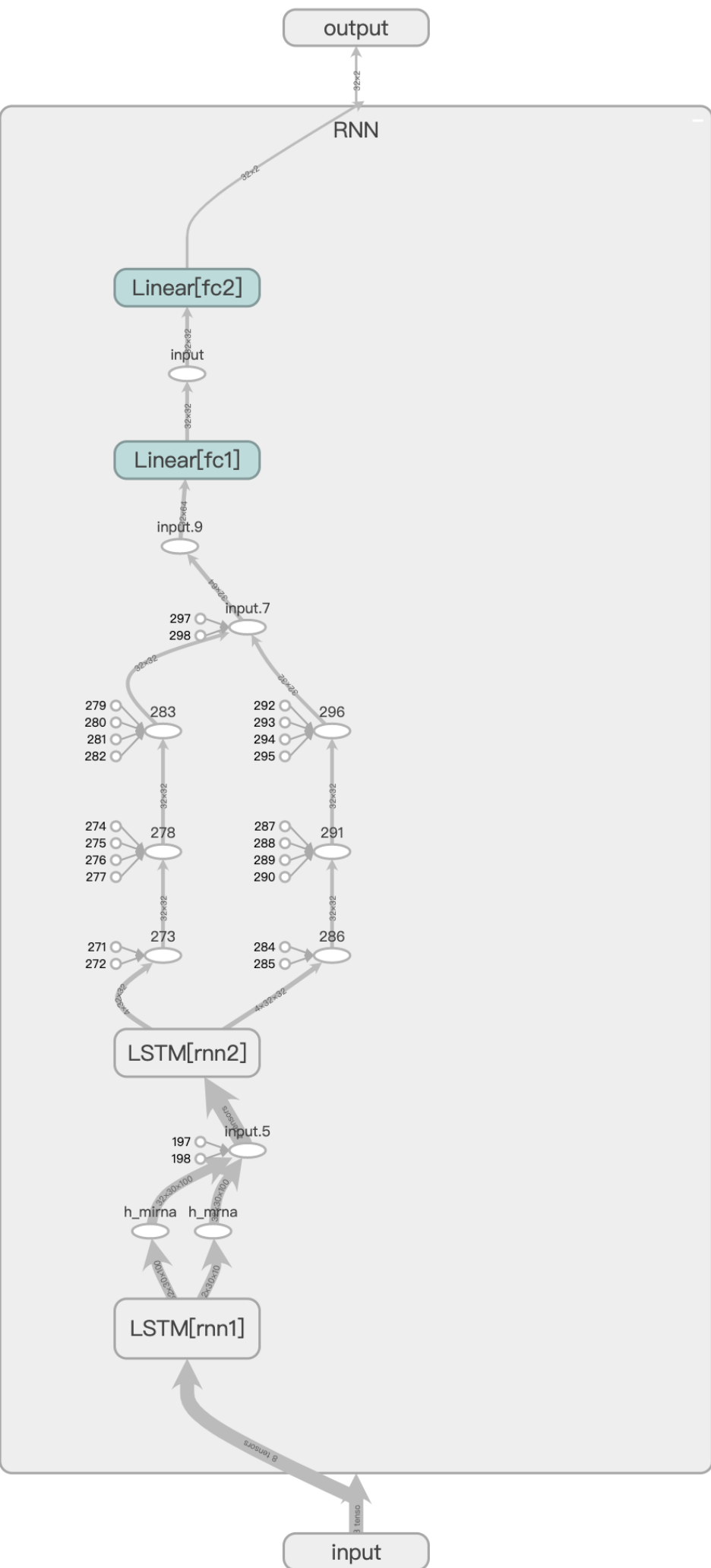

Supplement: Supplementary Materials — We used TensorBordX to record all parameter changes that the model went through from input to output, where RNN represents our deep learning model, LSTM (rnn1) represents the first layer of BiLSTM, LSTM (rnn2) represents the second layer of BiLSTM, Linear (fc1) represents the first linear layer, and Linear(fc2) represents the second linear layer. Figure S1: where RNN represents our deep learning model, LSTM (rnn1) represents the first layer of BiLSTM, LSTM (rnn2) represents the second layer of BiLSTM, Linear (fc1) represents the first linear layer, and Linear(fc2) represents the second linear layer. Figure S2: details of the internal parameters of the BiLSTM structure. [file 4490154.f1.zip › figure s1.pdf]

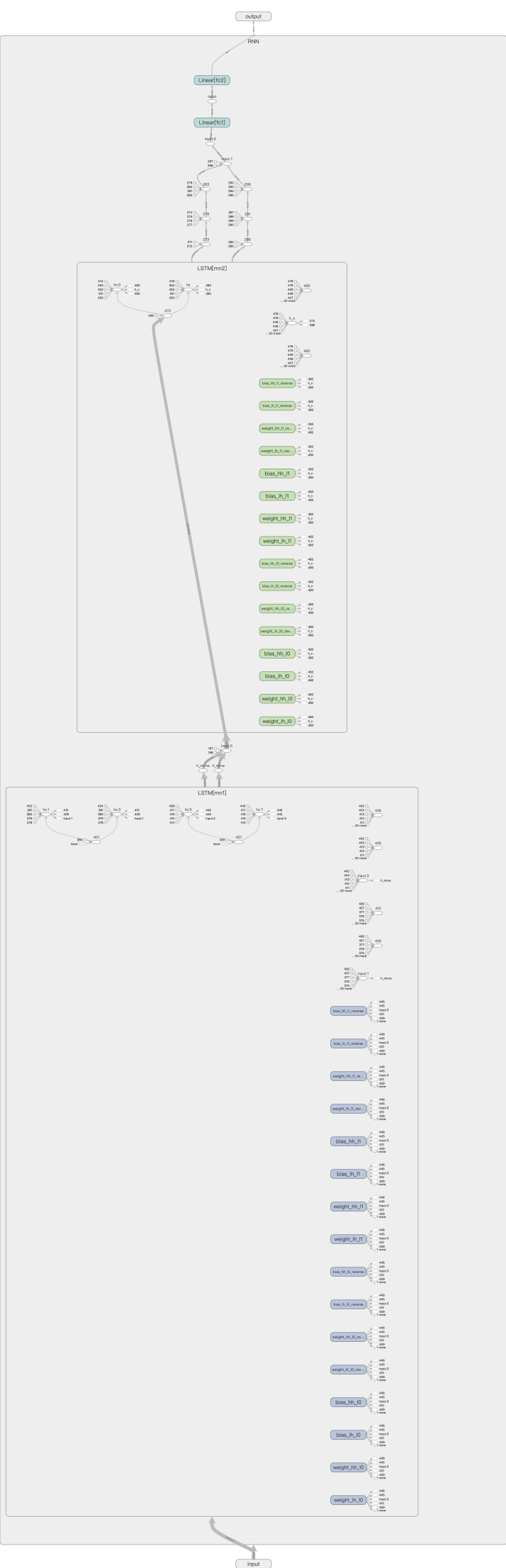

Supplement: Supplementary Materials — We used TensorBordX to record all parameter changes that the model went through from input to output, where RNN represents our deep learning model, LSTM (rnn1) represents the first layer of BiLSTM, LSTM (rnn2) represents the second layer of BiLSTM, Linear (fc1) represents the first linear layer, and Linear(fc2) represents the second linear layer. Figure S1: where RNN represents our deep learning model, LSTM (rnn1) represents the first layer of BiLSTM, LSTM (rnn2) represents the second layer of BiLSTM, Linear (fc1) represents the first linear layer, and Linear(fc2) represents the second linear layer. Figure S2: details of the internal parameters of the BiLSTM structure. [file 4490154.f1.zip › figure s2.pdf]
